# Tumor Immune Microenvironment and Immunotherapy in Brain Metastasis From Non-Small Cell Lung Cancer

**DOI:** 10.3389/fimmu.2022.829451

**Published:** 2022-02-17

**Authors:** Yuchang Wang, Rui Chen, Yue Wa, Shikuan Ding, Yijian Yang, Junbo Liao, Lei Tong, Gelei Xiao

**Affiliations:** ^1^ Department of Neurosurgery, Xiangya Hospital, Central South University, Changsha, China; ^2^ Diagnosis and Treatment Center for Hydrocephalus, Xiangya Hospital, Central South University, Changsha, China; ^3^ Hunan International Scientific and Technological Cooperation Base of Brain Tumor Research, Xiangya Hospital, Central South University, Changsha, China; ^4^ National Clinical Research Center for Geriatric Disorders, Xiangya Hospital, Central South University, Changsha, China; ^5^ Department of Neurosurgery, Affiliated Nanhua Hospital, Hengyang Medical School, University of South China, Hengyang, China

**Keywords:** cancer, BM, immunotherapy, NSCLC, PD-1 inhibitor, PD-L1 inhibitor

## Abstract

Brain metastasis (BM), a devastating complication of advanced malignancy, has a high incidence in non-small cell lung cancer (NSCLC). As novel systemic treatment drugs and improved, more sensitive imaging investigations are performed, more patients will be diagnosed with BM. However, the main treatment methods face a high risk of complications at present. Therefore, based on immunotherapy of tumor immune microenvironment has been proposed. The development of NSCLC and its BM is closely related to the tumor microenvironment, the surrounding microenvironment where tumor cells live. In the event of BM, the metastatic tumor microenvironment in BM is composed of extracellular matrix, tissue-resident cells that change with tumor colonization and blood-derived immune cells. Immune-related cells and chemicals in the NSCLC brain metastasis microenvironment are targeted by BM immunotherapy, with immune checkpoint inhibition therapy being the most important. Blocking cancer immunosuppression by targeting immune checkpoints provides a suitable strategy for immunotherapy in patients with advanced cancers. In the past few years, several therapeutic advances in immunotherapy have changed the outlook for the treatment of BM from NSCLC. According to emerging evidence, immunotherapy plays an essential role in treating BM, with a more significant safety profile than others. This article discusses recent advances in the biology of BM from NSCLC, reviews novel mechanisms in diverse tumor metastatic stages, and emphasizes the role of the tumor immune microenvironment in metastasis. In addition, clinical advances in immunotherapy for this disease are mentioned.

## Introduction

The rate of brain metastasis (BM) from solid tumors was about 7.3 persons/100,000 with most patients above 50 years (91.9%) who have a poorer prognosis. The most common primary lesions for BM are the lung (80%), melanoma (3.8%), breast (3.7%), and kidney/renal pelvis (3.0%) (1–3) (**Table 1**). Non-small cell lung cancer (NSCLC) accounts for almost half of all BM cases, and NSCLC is associated with a horrible prognosis (4, 5). Patients who do not receive therapy for BM from NSCLC have a median overall survival of only 1 to 2 months. Age, extracranial tumor activity, the number of BM, and the initial tumor type/molecular subtype are important factors determining patients’ prognosis (6, 7). Previously, surgical resection was a high-risk procedure that placed strict limits on the number and location of the lesions in the brain (8). Whole-brain radiotherapy and stereotactic radiotherapy, both effective therapies and local controls for BM from NSCLC, are other options (9). However, radionecrosis may occur in more than 1/3 of patients, resulting in neurotoxicity (10). On the other hand, chemotherapeutic drugs are often incapable of crossing the blood-brain barrier (BBB) and reaching intracranial lesions. As a result, the use of chemotherapeutic drugs to slow the course of the central nervous system(CNS) is less meaningful (11).

**Table 1:** Contrast of NSCLC BM, breast cancer BM, melanoma BM and primary brain cancer (Glioblastoma).

	NSCLC BM	breast cancer BM	melanoma BM	Glioblastoma
Median number of tumors per patient	3 (1-6)	3 (1-11)	2 (1-6)	1
Median single tumor volume in cc	0.06	0.01	0.12	43.20
Preference of distribution	the infratentorial area, frontal lobe	structures supplied by the posterior circulation areas	supratentorial area	supratentorial area (frontal, temporal, parietal and occipital lobes)
Common Mutations	EGFR, ALK, KRAS, RET, ROS-1	HER2, PR, ER	BRAF, NRAS	EGFR, PTEN, TP53, CDKN2A
Median OS in Months	5.0 、16	10.0-15.0	2.5-6.0	11.4-15
Median age of the patients (year)	62	48.8	60	64
Common biomarker in ICI	PD-L1	PD-L1	PD-L1, CTLA-4	None

NSCLC, non-small cell lung cancer; BM, brain metastasis; OS, overall survival; EGFR, epidermal growth factor receptor; ALK, anaplastic lymphoma kinase; KRAS, Kirsten rat sarcoma viral oncogene homolog; RET, rearranged during transfection; ROS-1, c-ros oncogene 1; HER2, Human epidermal growth factor receptor 2; PR, progesterone receptor; ER, estrogen receptor; BRAF, v-raf murine sarcoma viral oncogene homolog B1; NRAS, neuroblastoma RAS viral oncogene homolog; PTEN, phosphatase and tensin homolog; TP53, Tumor Protein P53; CDKN2A, Cyclin Dependent Kinase Inhibitor 2A.

The occurrence and BM of NSCLC are closely related to the tumor microenvironment (TME). TME is the surrounding microenvironment where tumor cells live. TME comprises cellular and non-cellular components such as blood vessels, fibroblasts, immune cells, bone marrow-derived inflammatory cells, signaling chemicals, and extracellular matrix (ECM) (12). Tumors and their surroundings are inextricably linked and constantly interact, with tumors influencing their microenvironment by releasing cellular signaling molecules that promote tumor angiogenesis and induce immune tolerance. So immune cells in the microenvironment influence cancer cells’ growth and development. The microenvironment of the brain is different and bears little resemblance to other organs. Even when BM occurs, the microenvironment remains distinctly different from other organs (13).

Cancer immunotherapy has become a significant study issue in recent years, spawning new techniques to modulate the immune system. BM comprises complicated immunological microenvironment and signaling processes, with many potential treatment targets still to be discovered (11). Evasion of the host immune response is a hallmark of cancer progression. Blocking of cancer immunosuppression by targeting immune checkpoints provides a suitable strategy for immunotherapy in patients with advanced cancers such as NSCLC and melanoma (14). Immune checkpoint inhibitors (ICIs) target inhibitory immune checkpoints and boost the anti-tumor immune response. ICIs have shown excellent clinical success in advanced melanoma, metastatic kidney cancer, and NSCLC. Even pembrolizumab alone has been reported to eradicate stage IV BM from NSCLC (15). Because the immune response in the brain is so tightly controlled, researching immunomodulatory therapy for BM is challenging.

This article discusses the impact of TME on NSCLC and BM, focusing on the specificity of BM. The presence of barriers and specialized cells makes the TME of BM unusual and demonstrates differences from NSCLC in many aspects, especially in the immune. Features of BM suggests a lack of research about the brain and directs therapy in a new direction. The birth of immunotherapy is both dependent on the gradual refinement of the theory and inextricably linked to the remarkable efficacy in practice. The excellent role played in patients with BM from NSCLC has also led to combining traditional approaches and immunotherapy.

## BBB

BBB is a selective barrier of CNS, which isolates the brain from the harmful substance from the systemic circulating blood, consisting of endothelial cells, pericytes, and the foot processes of astrocytes (16). BBB is the most important structure for the tumor cell to get through in BM. The key to the wholeness and selectivity of the BBB is the tight junction composed of claudins, occludins, zona occludens proteins, and junctional adhesion molecules (17).

The immunocytes are also involved in the destruction of BBB. T cell is founded as a helper of BBB crossing. In the research of Mustafa, Dana A M, et al., Guanylate-Binding Protein 1 is considered a vital T cell-induced protein, which promotes the tumor cells crossing the BBB. The patients who developed BM show Guanylate-Binding Protein 1 upregulate (18). In addition, as one of the most widely distributed cell types in the brain, astrocytes play an important part in the formation of BM, including BBB crossing. The astrocyte could secrete multiple matrix metalloproteinases (MMP), among which MMP2 and MMP9 are widely researched and considered a breaker of BBB (19). The research of Rempe et al. shows that MMP1 is strongly associated with BM by degrading Claudin and Occludin but not tight junction protein Zo-1 (20). Microglia is an immunocyte of the CNS, mediating apoptosis. However, when malignant tumors metastasize, they inhibit apoptosis and invade the BBB using tissue damage responses involving C-X-C chemokine receptor (CXCR)4 and *Wnt* signaling (21). According to the research of Qiao S et al. in melanoma, the microglia is found highly expressed MMP3, relating to the decrease of Zo-1, which may facilitate tumor invasion (22). The previous studies have mainly focused on breast cancer and melanoma, but the differences between tumor cell types are still unknown. Due to the importance of the BBB and its extensive interactions with surrounding cells and stroma, the study of the BBB is essential before further description of the development of BM. The role of BBB in the development of BM from NSCLC will be further described in the following section through the process of BM.

## Development of BM From NSCLC

**Figure 1:**
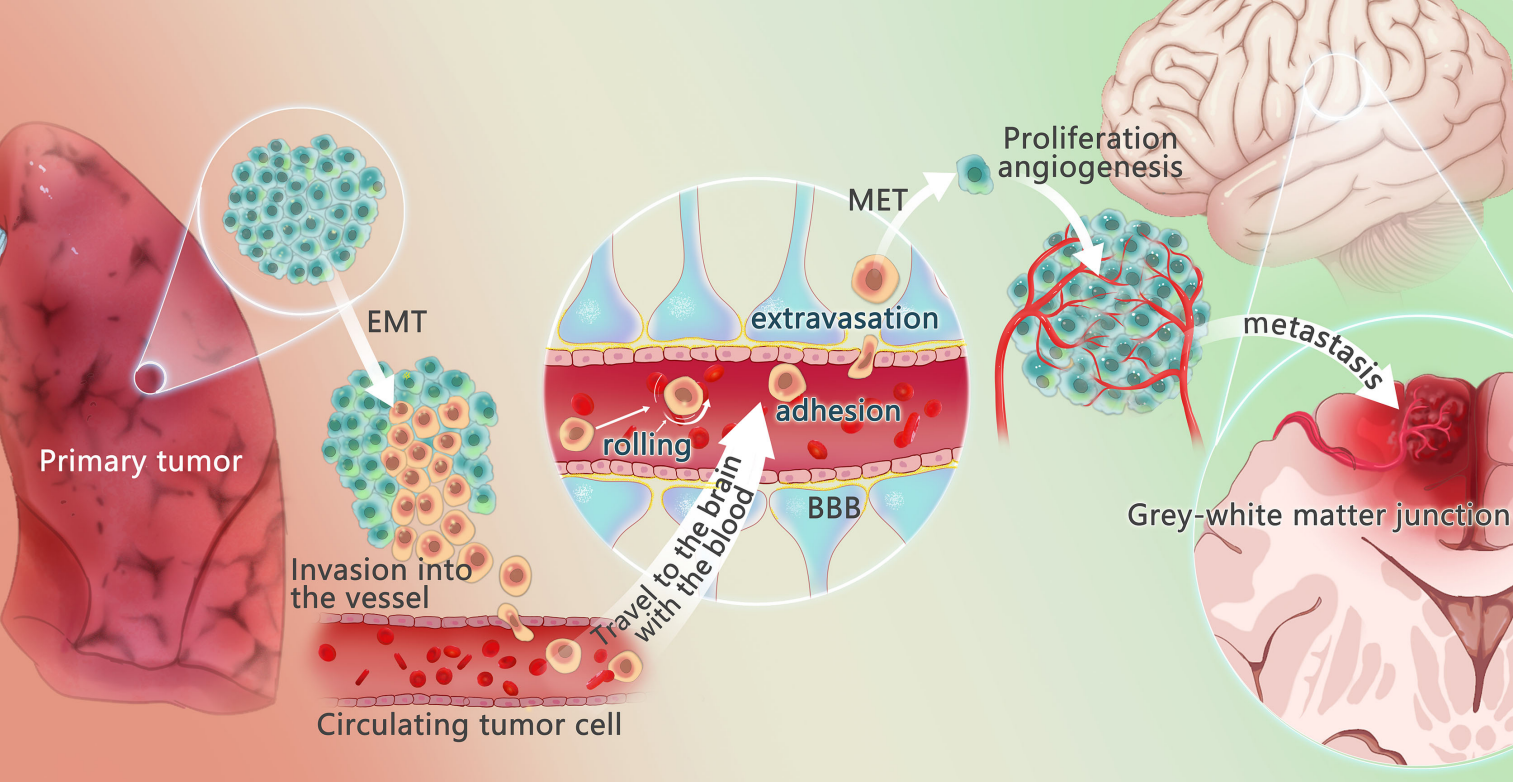
Mechanism of lung cancer brain metastasis. The changes of tumor microenvironment may promote some tumor cells to undergo epithelial-mesenchymal transition (EMT) process, to get the potential of metastasis and avoid apoptosis. These tumor cells escape from the primary tumor in the lung, invade the vessels, and circulate through the vessels, called circulating tumor cells (CTCs). Under the action of chemokines, CTCs reach the brain, cross the blood-brain barrier through rolling, adhesion and extravasation under the effect of E-ligand and integrin, undergo mesenchymal-epithelial transformation (MET) to regain the characteristic of the primary tumor, recover the characteristics of the primary tumor, and produce and adapt to the new tumor microenvironment. Angiogenesis is required for the growth of metastases. When pure oxygen diffusion is not sufficient for the tumor, the tumor gradually develops a hypoxic microenvironment and overexpress angiogenesis-stimulating factors, promoting the angiogenesis. The brain metastasis often happens in the gray and white matter junction and vascular border zones, where there is a longer mean transit times (MTT)of blood flow, providing more chance to overcome the blood-brain barrier.

The development of BM from NSCLC is a complicated, interacting, and changing process that involves tumor cells (mutations, epigenetic variation), cytokines, interactions with other tumor cells, and normal cells in the surrounding area. Clinical identification of high-risk BM in individuals with NSCLC is critical. As a result, a thorough understanding of NSCLC and BM is required. Since 1889, Paget introduced the “seed and soil” theory to explain BM (23), epithelial-mesenchymal transition (EMT), cancer stem cells, circulating tumor cells (CTCs), and other hypotheses have been proposed. Tumor metastasis mechanisms have steadily been added. The development of BM from NSCLC is closely related to the TME (11), which is involved in these mechanisms (**Figure 1**)

EMT is a fundamental notion in the development of metastasis. Most tumors originate from epithelial cells and spread into the surrounding connective tissue (cancerous mesenchymal cells). Mesenchymal cells, unlike epithelial cells, are rarely involved in intercellular interactions and lack cell polarity. Mesenchymal cells are more aggressive than epithelial cells because of these properties, and EMT is required to transform benign tumors into invasive carcinomas (24). The existence of EMT is demonstrated by abnormal expression of N-cadherin in tumor metastasis (25), and the transcription factor Snail, which regulates N-cadherin, is implicated in the migration and invasion of NSCLC cells mediated by Zinc transporter4. In the Snail-N-cadherin signaling axis, Zinc transporter4 plays a crucial regulatory role in encouraging NSCLC progression, and it may be a prognostic marker and treatment target for NSCLC (26). Hypoxia has also been identified as a critical cause of EMT in NSCLC. Hypoxia-inducible factor (HIF) is induced to activate in primary TME as the oxygen supply at the tumor’s center decreases with tumor growth. HIF-1α, which elevates midkine (MDK) levels in NSCLC cells, is required for NSCLC cell proliferation in hypoxic environments. *Via* paracrine signaling, MDK stimulates endothelial cell migration and neointima formation. MDK produced by NSCLC cells also interacts with Notch2, activating *Notch* signaling, inducing EMT, upregulating NF-κB, and promoting cancer (27). MDK inhibitors could be a new treatment option for NSCLC patients. Angiogenesis and lymphangiogenesis are triggered by hypoxia, growth hormones, and receptors in the TME, which enhance cancer cell dispersion.

Circulating tumor cells, also known as CTCs, are injected into the local blood and lymph and survive the dissemination process. Tumor cells are first isolated from the primary tumors and spread throughout the surrounding tissues, capillaries, tiny veins, and lymphatic system. Tumor cells are vulnerable to physical and immunological damage once in circulation, making it harder for CTCs to launch a metastatic cascade response. To overcome the difficulties, tumor cells must boost the programming and intrinsic adaptation required for metastasis. Every CTC must get through the BBB to reach the brain. It is the first step to overcome the BBB for the CTCs to get to the brain and propagate. Several experiments have shown that the CTCs could traverse the BBB. However, the specific mechanism is not well understood yet (28). There are still some cells that succeed in overcoming the BBB. The tight junction of BBB makes it harder to pass than the other organs. Several cytokines, chemokines, and inflammatory agents can disrupt these interactions. Over 70% of BM shows varying degrees of BBB destruction, implying the CTCs might affect the tight junction (29).

The step CTCs crossing the BBB is like the leukocyte (rolling, adhesion, extravasation). Two possible pathways of extravasation have been described: the paracellular route and the transcellular route (30, 31). CD15 and CD15s interact with CD62E to promote lung cancer cell adherence to the brain endothelium, exhibiting features comparable to white blood cells but interfering with FUT4/CD15 and FUT7/CD15s can inhibit CTCs invasion (32–34). As reported, FUT4 has been linked to programmed cell death protein 1 (PD-1)-related immunosuppression, which leads to poorer prognosis in patients with operable lung cancer (35). CTCs could produce many factors to help destroy the BBB, including cyclooxygenase cyclooxygenase2, C-X-C motif chemokine ligand (CXCL)12/CXCR4, ST6GALNAC5, cathepsin S, MMP-1, α-crystallin, angiopoietin-like 4, and vascular endothelial growth factor (28, 36, 37). The link between CTCs and BBB is reciprocal, which frequently exhibits positive feedback. For example, when metastatic tumor cells and tumors penetrate brain tissue, the integrity of the BBB is destroyed, and the degradation of BBB integrity further promotes tumor BM.

## TME and Immunotherapy of BM from NSCLC

**Figure 2:**
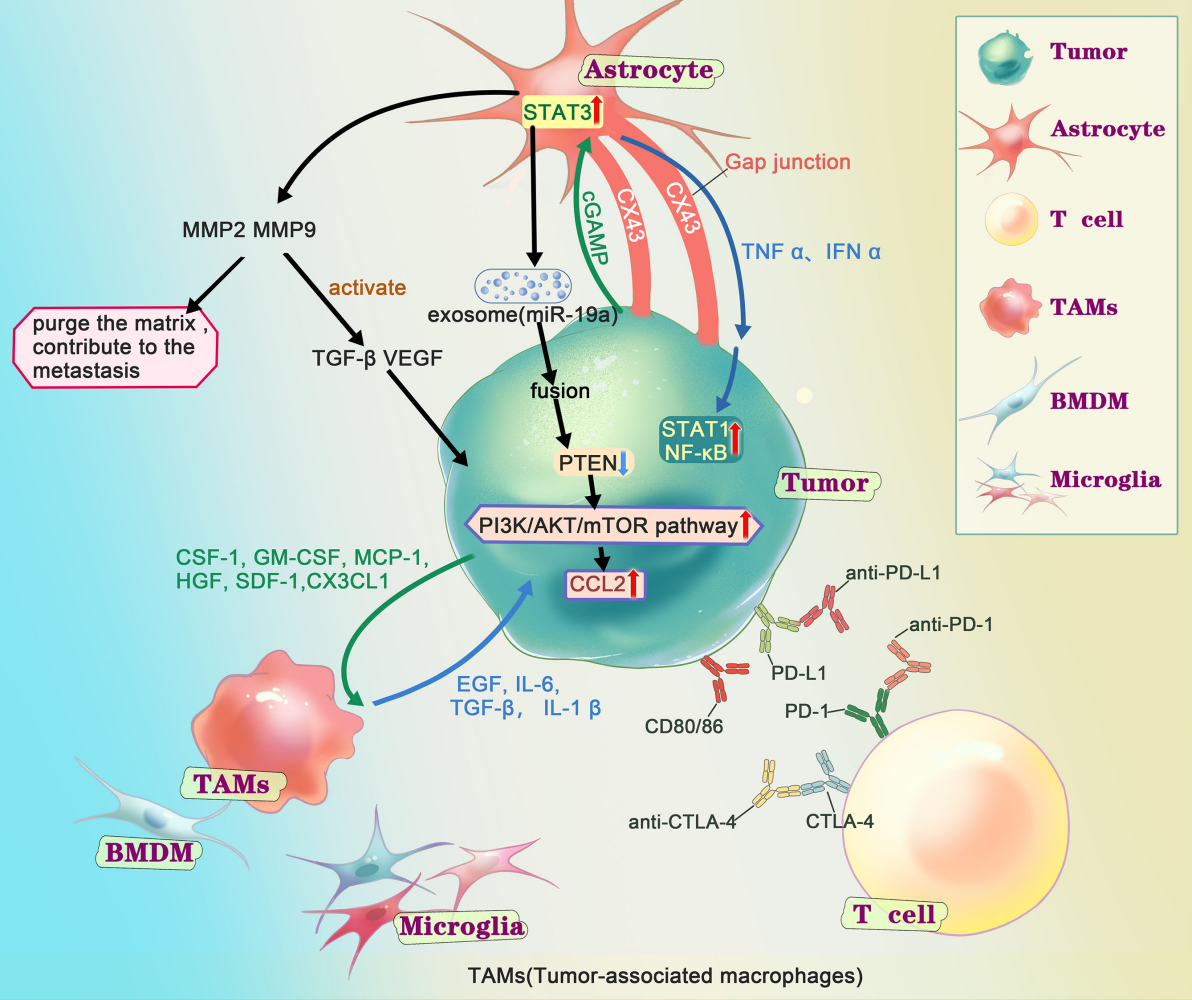
Interactions of metastatic tumor cells with the brain microenvironment. After the tumor cells get into the CNS (central nervous system), according to the “seed and soil”, series of interactions happened between the tumor cells and the microenvironment in brain. Extensive research has shown that astrocytes play an important role in metastasis through matrix metalloproteinase 2 (MMP2), MMP9, exosome and gap junction, generally supports the growth and invasion. MMP2、MMP9 are associated with tumor growth. On the one hand, it could degrade components of the extracellular matrix and basement membrane, on the other hand, it helps to activate TGF-β and VEGF. Several studies have shown that after fusion of AST-generating exosomes with tumor cells, miRNAs contained in the exosomes cause tumor cells to under-express PTEN and further activate the PI3K/AKT/mTOR pathway, leading to more chemokine ligand 2(CCL2), promoting the growth of tumor. There are gap junctions composed of connexin 43 (Cx43) between tumor cell and astrocyte. Through the gap junction, astrocyte release cytokines such as IFNα and TNFα, activating STAT1 and NF-κB pathways, supporting tumor growth. At the same time, tumor cell could transfer cGAMP to astrocytes, activate the STAT3 pathways. Tumor-associated macrophages and microglia (TAMs), including bone marrow-derived macrophages (BMDMs) and microglia (MG), secret growth factors (EGF, IL6, TGF-β, IL-1β), contribute to the colonization. And the tumor cells release chemokines and cytokines (CSF-1, GM-CSF, MCP-1, HGF, SDF-1, CX3CL) to recruit TAMs towards the tumor cells. There are immune checkpoints expressed on T-cells, such as PD-1 and CTLA-4, which tumor cells could bind and deactivate T-cells, suppressing anti-tumor immunity. Checkpoint immunotherapy use antibodies to inhibit immune checkpoint to prevent the T-cell from deactivation and control the tumor.

The TME of BM is not identical to that of primary NSCLC (13). In fact, in the physiological scenario, normal brain tissue differs from other tissues by a few key characteristics. The ECM composition and type of tissue-resident cells (including microglia, astrocytes, and neurons) in normal brain tissue are distinct. The brain is physically protected by the BBB, which plays an important role in the immune response (38). In the event of BM, the metastatic TME in BM is composed of ECM and tissue-resident cells that change with tumor colonization, as well as blood-derived immune cells. Even though the concept that the brain is an “immune privileged” organ was widely recognized, it is now widely believed that immune cells from the peripheral circulation can penetrate the skull (39). This change originates from the discovery of meningeal lymphatics and the hypothesis that BM might compromise the integrity of the BBB (40). The immune microenvironment of BM undergoes profound changes with the alteration of native immune cells in the brain and the entry of blood-derived immune cells, and these changes are often immunosuppressive (38) (**Table 2** and **Figure 2**).

**Table 2:** Feature of the TME of NSCLC brain metastasis comparing with primary tumor.

Microenvironment features		Significance
T cells	Fewer T cells in total (including Th1 cells or TILs)	Form an immunosuppressed TME, providing a better chance for the tumor growth
	Lower abundance measurements of Th1 or CD8 T genes	
	Inhibition of Th1 immune response	
Macrophage	Higher Gene expression levels of the macrophage (M2-like) marker arginase-I (ARG1)	Present an immunosuppressed TME
	more anti-inflammatory TAMs	
	Lower relative abundances of macrophage genes	
DC	Inhibition of DC maturation	Suppress antigen presentation
	Lower relative abundances of DC genes	
VCAM1	Reduced expression of VCAM1	Suppress adhesion of leukocytes
Astrocytes	pSTAT3^+^ reactive astrocytes	Inhibit CD8^+^ T cell activation and increase expression of immuno-inhibitory protein PD-L1, promote brain metastasis
	EVs or gap junctions with tumor cell	Promote the survival of metastasis and increase the resistance of chemotherapy

TME, tumor microenvironment; NSCLC, non-small cell lung cancer; type 1 T helper; TILs, tumor Infiltrating Lymphocytes; TAMs, tumor associated macrophages; DC, dendritic cell; VCAM1, vascular cell adhesion molecule 1; pSTAT3, Phosphorylated transducer and activator of transcription-3; PD-L1, programmed death ligand 1; EVs, extracellular vesicles.

### T Cells and Regulatory T Cells

Regulatory T (Treg) cells are primarily CD4+ T cells that constitutively express CD25 molecules and specifically express foxp3 transcription factors. There is growing evidence for Treg cells’ active involvement in the negative regulation of various physiological and pathological immune responses (41). Mature Treg cells are generated through two distinct developmental processes, CD25 + Treg cell progenitors and Foxp3lo Treg cell progenitors. In either developmental process, Treg cells are dependent on IL-2 for development and maintenance of activity (42). Different evidence exhibits different specialties of Treg from the two sources, with mature Treg cells from the former having a unique position in promoting immune tolerance and the latter having a unique role in preventing autoimmunity (43). When NSCLC occurs, T Cells and Treg cells keep the TME of BM in an immunosuppressed state. Many Treg cells infiltrating, especially a low CD8 + T cell to Treg cell ratio in tumor tissue, is significantly associated with poor prognosis (44). When BM occurs in NSCLC, the abundance and infiltration of CD8+ T cells, CD4+ T cells, and Treg cells in the microenvironment of BM are lower compared to the primary focus (45, 46). And this relatively low value does not involve changes in T cell sequences, with no statistically significant differences in T-cell receptor β sequence clonality in BM and primary lung tumor and non-malignant lung parenchyma (47). More reduced T cell abundance and infiltration compared to NSCLC, along with suppression of antigen presentation (suppressed dendritic cells maturation), lymphocyte extravasation, and leukocyte adhesion (reduced vascular cell adhesion protein 1), together contribute to an immunosuppressive microenvironment in BM (47).

Tumor-infiltrating Treg cells expressed higher surface molecules related to T cell activation in CD25, T-lymphocyte-associated protein-4 (CTLA-4), PD-1, and TNF receptor superfamily members compared to Treg cells in lymphoid tissue, non-lymphoid tissue, or blood (48). CTLA-4 and PD-1 belong to the second signal, which comes after the initial signal to activate CTL in cancer immunity. In tumor immunity, the initial signal is T cell receptor recognition of the major histocompatibility complex accompanied by cancer-specific antigens. The second signal is often known as immunological checkpoints, including stimulatory and inhibitory molecules that keep the immune system active (49). Tumor cell invasion is suppressed after T cell activation. Cancer progression is characterized by the evasion of the host immune response. As a result, blocking cancer immunosuppression can occur throughout the entire process of immune activation, in which targeting immunological checkpoints is an effective immunotherapy method for BM. In BM from NSCLC, CTLA-4 has been less studied, while PD-1 has been more studied and related therapies have been developed. The primary physical role of PD-1 is to maintain peripheral tolerance and keep T cell responses within the appropriate physiological range (50). Because an immune response triggers the PD-1/programmed cell death ligand 1(PD-L1) regulatory mechanism, a negative feedback loop that reduces local T-cell reactions and tissue damage is created (51). When NSCLC occurs, tumor-derived IL-6 stimulates PD-L1(+) myeloid cells (52). When PD-1 binds to PD-L1, cytotoxic T cells that detect tumor cells become inactive, promoting immune escape (14).

As a result, the expression of PD-1/PD-L1 is one of the most critical indicators of tumor status (53). ICIs may be helpful in the treatment of individuals with NSCLC to prevent BM, as impaired peripheral immune function increases tumor spread. When BM occurs in NSCLC, activated T cells, monocytes, and dendritic cells are among the immune cells that express PD-1 (54). Meanwhile, tumor cells, as well as immune cells, such as T cells, B cells, macrophages, and dendritic cells, express PD-L1 (55). Due to differences in TME, there may be inconsistent responses to ICIs for NSCLC between primary tumor lesion and BM. On the one hand, PD-1 + tumor infiltrate lymphocytes (TILs) are less permeable in BM, which may significantly reduce the ICIs effect of anti-PD-1 (45). On the other hand, PD-L1 is generally higher at metastatic sites (56), which is also associated with the efficacy of ICIs. PD-1/PD-L1 expression in patients with BM from NSCLC taking immunotherapy may predict survival independently (57).

Based on the functional role of PD-1/PD-L1 expression and distribution, it is unavoidable to predict its illustrious future in treating BM from NSCLC. Both PD-1 and PD-L1 inhibitors were beneficial in patients with CNS diseases in the first trial focusing on a group of patients with BM from NSCLC and assessing the effects of immunotherapy (58). Despite this, the mechanism of action of PD-1/PD-L1 is not entirely clear (38), and drug development is underway, with more research as a goal in the future.

### Tumor‐Associated Macrophages and Microglia

Tumor‐associated macrophages and microglia (TAM/M) are highly plastic and receptive to cytokine, chemokine, and growth factor signals. Their activation promotes cell invasion, angiogenesis, metastasis, and immunosuppression (59). TAM/M is the main non-tumor cell in glioma (60) and is second in density to BM lymphocytes (61). TAM/m activation types include a pro-inflammatory, anti-tumor M1-like phenotype and an anti-inflammatory, pro-tumor M2-like phenotype. M1 macrophages highly express inducible nitric oxide synthase and secrete IL-1, IL-12, NO, and TNF-α, which have tumor-killing effects. M1-type markers are TNF-α, CD80, CXCL9, and CXCL10 (62). In response to IFN-γ and lipopolysaccharide, M1 macrophages, which secrete high levels of IL-12, inhibit tumor development (63).M2-type macrophages secrete arginase, IL-10, lipopolysaccharide, IFN-γ, inhibit CD8+ T cell proliferation, and promote tumor cell growth.M2-type markers CD204 and CD206, CCL17 and CCL18, etc. (62). M2 macrophages are exposed to IL-4 or IL-13 and produce immunosuppressive substances (IL-10 and TGF-β) that promote tumor growth by increasing STAT3 expression and inhibiting antigen presentation (63, 64).

In BM, the proportion of most immune cell subpopulations decreases, but with a few exceptions, such as M2-macrophages, whose ratio in the proportion is strongly associated with BM (65). Therefore, the change of TAM/m from M1 to M2 phenotype may be one of the reasons for the shift of TME from anti-tumor to pro-tumor (66). Recent in-depth studies of lung cancer BM have revealed that around 10% of TAM/m contain peripheral-derived macrophages in early metastases, rising to 20% in massive metastases (67). In contrast to primary NSCLC, BM microenvironment macrophages infiltrate more and show elevated monocyte-derived macrophages (47). The phenomena may reflect the antitumor effect of TAM/m in the early stages of BM formation and the accumulation and activation of TAM/m in the later stages (66).

C-X3-C motif chemokine receptor 1/C-X3-C motif chemokine ligand 1 axis is a signaling pathway which regulates TAM/m (66). This pathway has been much studied in brain inflammation, and its activation regulates microglia activation in a quiescent state (68), thereby inhibiting the release of IL-6, TNF-α, etc. (69). The relationship between this pathway and BM is inconclusive and warrants investigation. Considering the complex involvement of C-X3-C motif chemokine receptor 1 in the metastatic cascade, its nonspecific inhibitors may not be an appropriate therapeutic option (66). In addition, colony-stimulating factor 1 receptor inhibitors acting on TAM have shown promise in melanoma and enhancing chemotherapy response and might be applied in BM (70). Overall, immunotherapy against TAM/m includes both modulation of TAM/M polarization and inhibition of the survival and function of TAM/M. Some of the mechanisms have been studied to some extent, but there is still a lack of clinical studies demonstrating the actual therapeutic effects of BM from NSCLC targeting these mechanisms.

### Astrocytes

Astrocytes are one of the most abundant cell types specific to the CNS and play an essential role in mediating tissue-specific communication in the brain (39). Also, astrocytes are the most abundant glial cells in the TME of BM. After the injury, astrocytes alter their phenotype, inducing a transcriptional program known as reactive astrocyte proliferation (71). Reactive astrocytes, like microglia, have two faces. When in contact with tumor cells early enough, astrocytes maintain microenvironmental balance and trigger apoptosis, inhibiting metastatic colonization. However, with further interactions (72), the shift from a metastatic suppressive to a facilitative milieu due to the intricate interplay between astrocytes and microglia is created (39). The effects between tumor cells and reactive astrocytes are mutual. IL-8, macrophage movement inhibitory factor, and fibrinolytic enzyme activator inhibitor 1 secreted by metastatic lung cancer cells can activate astrocytes that produce growth factors (IL-6, IL-1, and TNF-α), encouraging tumor growth (72). Also, astrocytes express a significant variety of immunosuppressive molecules (IL-10, TGF-β, etc.) that influence innate and adaptive immune cells (39).

When infiltrating lung cancer cells are exposed to astrocytes, plasmin from the ECM is a defense against metastasis. Plasmin inhibits BM by converting astrocyte FasL into a paracrine death signal and inactivating the axon guidance molecule L1CAM. In contrast, plasminogen activator inhibitory serpins secreted by tumor cells are a barrier against plasmin (73). Once bound to the astrocyte gap junction network, CTCs use connexin 43 to transfer the second messenger cGAMP to astrocytes. Thus, the *STING* pathway is activated to produce inflammatory cytokines such as IFN-α and TNF-α (74), which act as paracrine signals to promote tumor growth. So, we know the principle that drugs working on gap links can treat established BM. In addition, astrocytes fight BM by exogenously secreting miRNA-142-3p, mediating the downregulation of transient receptor potential ankyrin-1. In BM from lung adenocarcinoma, the downregulation inhibits transient receptor potential ankyrin-1-mediated activation of fibroblast growth factor receptor 2 and impedes metastasis (75). In the BM of NSCLC, we suggest that reactive astrocytes contact with tumor cells after extravasation and produce plasmin as a defense against metastasis at first. In contrast, tumor cells build proteins, such as plasminogen activators, that allow them to survive despite the production of plasmin. At the same time, reactive astrocytes relax endothelial cell junctions by cytokine and regulate tumor cell gene expression by exosomes, thus influencing the progression of progressive BM around the lesion (76).

### Cancer-Associated Fibroblast

The role of cancer-associated fibroblasts (CAFs) in the microenvironment of NSCLC BM remains to be explored. Evidence of fibroblastic entities and their presence in the brain is limited, though fibroblasts are linked to the progression and metastasis of many cancers (77) for their pro-angiogenic and immunosuppressive effects. CAFs are activated fibroblasts in TME that release a significant number of cytokines and chemokines that affect a variety of leukocytes, including CD8+ T cells, Treg cells, and macrophages (78), playing effects such as pro-angiogenesis and immune suppression. CAFs express fibroblast activation protein-α, whose immunosuppression is mediated by CXCL12. This chemokine attaches to cancer cells and rejects T cells *via* a process dependent on signaling by the CXCL12 receptor CXCR4 (79).

### TME Difference Between BM From NSCLC and Other Brain Tumors

The TME in BM is different from the extracranial primary tumor. However, BM’s molecular and genetic features often correlate with primary brain malignancies such as glioblastoma, some of which don’t exist in the primary tumor (80). For example, EGFR-amplified BM from NSCLC shows similar oncogenic changes with EGFR-amplified classic glioblastoma and Her2+ breast cancer BM (81). The similarity may be an adaptive mechanism for BBB selectivity. At the same time, molecular features may regulate TME, which means the similarity in oncogenic changes also lead to the similarity in TME (82).

## Recent Advancements in the Treatment of BM From NSCLC With Immunotherapy

Because of the unique anatomical and physiological properties of the CNS, current therapeutic methods for BM from NSCLC are restricted, and the prognosis is poor. Therefore, immunotherapy has been increasingly popular in the treatment of NSCLC. Some patients with NSCLC may benefit from ICIs (83). Intracranial and extracranial lesions may vary considerably in response to systemic immunotherapy due to genetic differences between BM and primary tumor, as well as TME variations (84) (**Table 3**)

**Table 3:** Update of immunotherapy in NSCLC BM.

Study	Therapy tested	Control or comparator therapy	Number of patients	Median OS (months)	Median PFS (months)	Other outcomes
Marcus Skribek et al. (85)	ICI	None	51	5.7	1.9	iPFS=2.5months
Guowei Zhang et al. (86)	Nivolumab	None	With BM: 32;	14.8	2.8	ORR=20.0%; DCR=53.1%; DOR=9.8 months
			Without BM: 41	20.2	4.9	ORR=19.5%;DCR=56.1%;DOR=28.8 months;
Konstantinos Rounis et al. (87)	PD-1/PD-L1 inhibitors	None	24	6.77	Not reported	Median duration of intracranial response (months): 7.53 Intracranial TTP (months): 4,3
Kazushige Wakuda et al. (88)	Pembrolizumab	None	With BM: 23;	21.6	6.5	ORR: 57%
			Without BM: 64;	24.6	7.0	ORR: 42%
Sarah B Goldberg et al. (58)	Pembrolizumab	None	37	9.9	1.9	Median TTP=1.8 months; RR=29.7%;
Lucio Crinò et al. (89)	Nivolumab	None	409	8.6	3.0	ORR: 17%; DCR: 39%; One year PFS: 20%; One year OS: 43%;
Diego Cortinovis et al. (90)	Nivolumab	None	37	5.8	4.9	ORR: 19%; DCR: 49%; OS rate at 1 year: 35%; PFS rate at 1 year: 31%;
Clément Gauvain et al. (91)	Nivolumab	None	43	Not reached	3.9	Intracerebral DCR: 51%;
Anna Cho et al. (92)	GKRS + ICI	GKRS alone	GKRS alone: 286;	5.6	Not reported	none
			GKRS+ICI: 82	24.2		
Matthew J Shepard et al. (93)	ICI+SRS	SRS alone	ICI+SRS: 17	Not reached	Not reported	12-month CR: 84.9%; rate of peritumoral edema progression: 11.1%
			SRS alone: 34	15.9		12-month CR: 76.3% rate of peritumoral edema progression: 21.7%
Charu Singh et al. (94)	anti-PD-1 therapy + SRS (ICI group)	chemotherapy (CT) + SRS (CT group)	ICI group: 39	10	Not reported	Median times to initial response: 49; Median time to maximal response: 105
			CT group: 46	11.6		Median times to initial response:84; Median time to maximal response: 182
Linda Chen et al. (95)	SRS + non- concurrent ICI; SRS + Concurrent ICI;	SRS alone	SRS alone: 181 (NSCLC 79%);	12.9	3.7	Mean number of new metastases: 4
			SRS + non- concurrent ICI: 51 (NSCLC 69%);	14.5	2.3	Mean number of new metastases: 4
			SRS + Concurrent ICI: 28 (NSCLC 7%);	24.7	2.3	Mean number of new metastases: 2
Chenglong Sun et al. (96)	ICI + chemotherapy; ICI + anti-angiogenic therapy	ICI alone	ICI alone: 30	27.43	Not reported	Combination therapy produced better survivals than monotherapy
			ICI + chemotherapy: 29			
			ICI + anti-angiogenic therapy: 10			
Muhammad Zubair Afzal et al. (97)	carboplatin/pemetrexed plus pembrolizumab (Cohort B)	carboplatin/pemetrexed (Cohort A)	Cohort A: 12	Not reported	4.1	ORR:58.3%; DCR: 75%; Median time to achieve response: 1.67 months;
			Cohort B: 5	Not reported	Not reached	ORR:80%; DCR: 80%; Median time to achieve response: 1.1 months;
Steven F Powell et al. (98)	Pembrolizumab + Platinum-Based Chemotherapy	Chemotherapy alone	Pembrolizumab + chemotherapy: 105	18.8	6.9	Incidences of treatment-related adverse events: 88.2%
			Chemotherapy alone: 66	7.6	4.1	Incidences of treatment-related adverse events: 82.8%

NSCLC, non-small cell lung cancer; BM, brain metastasis; OS, overall survival; PFS, progression-free survival; ICI, immune checkpoint inhibitor; iPFS, intracranial progression-free survival; ORR, objective response rate; DCR, disease control rate; DOR, duration of response; PD-1, programmed death 1; PD-L1, programmed cell death ligand 1; TTP, time to progression; RR, response rate; GKRS, gamma knife radiosurgery; SRS, stereotactic radiation surgery; CR, control rate; DBF, distant brain failure.

The role of ICIs in BM has been studied in retrospective analysis, case series, and preliminary clinical trials. These discoveries and their quick translation into clinical practice are causing significant shifts in treating patients with BM from NSCLC (85). Multiple studies have consistently shown that ICIs improve overall survival in both primary tumors and BM, supporting the utility of ICIs in the treatment of BM (88). PD-1 and PD-L1 are the most clinically relevant immune checkpoints. Several medications have been approved for treatment in patients with lung cancer BM, including anti-PD-1 drugs such as pembrolizumab and nivolumab, and anti-PD-L1 drugs like atezolizumab (89).

Pembrolizumab has been demonstrated to generate CNS responses at rates comparable to extracranial malignancies, with the overall survival rate in NSCLC patients exceeding the historical rate of BM (87). Nivolumab is a complete human IgG4 ICI, which binds to PD-1 on activated immune cells, breaking the PD-L1 interaction and increasing the anti-tumor response (86). Studies have shown Nivolumab to have a potential therapeutic effect on BM (90, 91). Atezolizumab monoclonal antibody has also been proven to extend the life expectancy of individuals with BM.

ICIs have changed the way advanced cancer patients are treated. Despite the positive results of ICI therapy, only a tiny percentage of patients responded, severely limiting the growth of ICI therapy. As a result, immunotherapy for BM from NSCLC is being developed with other combination therapies, such as radiotherapy or chemotherapy (92–95). Recent research has discovered that cancer patients having radiotherapy and/or chemotherapy have varied immunogenicity patterns, which could improve the function of immunotherapy, promote efficiency, and reduce side effects (96–98). Because of their potential to influence innate and adaptive immunity, certain chemotherapeutic drugs, such as cyclophosphamide, may improve the anticancer efficacy of immunotherapy.

## Conclusion

The significance and distinctiveness of BM compelled us to investigate it, and the TME is an essential research direction. NSCLC is the most prevalent tumor that causes BM, and while BM has a distinct TME, there are some similarities with original foci, such as the role of HIF. When BM occurs, the presence of BBB, as well as different types of ECM composition and tissue-resident cells, leads to the infiltration of blood-derived immune cells and the alteration of ECM and tissue-resident cells with tumor colonization, resulting in a distinct TME compared to primary NSCLC. Infiltration and phenotypic shifts of immune-related cells, in particular, cause alterations in signaling molecules that gradually transform the TME of BM from tumor rejection to growth. Because of these fundamental alterations, new ideas for treating BM from NSCLC emerged, and targeted immunotherapy was successfully implemented in practice. And the combination of conventional therapy and immunotherapy has begun. We feel that the importance of the TME is self-evident throughout the process, and it has facilitated the practice of immunotherapy. As a result of more in-depth research on the mechanism, treatment will be more comprehensive, and the prognosis of BM from NSCLC will be more encouraging.

## Author Contributions

YCW, YY, SD, and RC collected the related paper. LT, RC, YCW, SD, YW, and JL drafted and revised the manuscript. GX participated in the design of the review and helped to draft and revise the manuscript. All authors read and approved the final manuscript.

## Funding

This work was supported by National Natural Science Foundation of China (No. 82171347), the Scientific Research Project of Hunan Provincial Health Commission of China (No. 202204040024), and the Students Innovations in Central South University of China (No. S2021105330599, No. 2021105330046, No. XCX2021035).

## Conflict of Interest

The authors declare that the research was conducted in the absence of any commercial or financial relationships that could be construed as a potential conflict of interest.

## Publisher’s Note

All claims expressed in this article are solely those of the authors and do not necessarily represent those of their affiliated organizations, or those of the publisher, the editors and the reviewers. Any product that may be evaluated in this article, or claim that may be made by its manufacturer, is not guaranteed or endorsed by the publisher.

## Abbreviations

BM, brain metastasis; NSCLC, non-small cell lung cancer; BBB, blood-brain barrier; CNS, central nervous system; TME, tumor microenvironment; ECM, extracellular matrix; ICIs, immune checkpoint inhibitors; MMP, matrix metalloproteinases; CXCR, C-X-C chemokine receptor; EMT, epithelial-mesenchymal transition; CTCs, circulating tumor cells; MDK, midkine; PD-1, programmed cell death protein 1; CXCL, C-X-C motif chemokine ligand; Treg, regulatory T; CTLA-4, T-lymphocyte-associated protein-4; PD-L1, programmed cell death ligand 1; TILs, tumor infiltrate lymphocytes; TAM/M, tumor-associated macrophages and microglia; CAFs, cancer-associated fibroblasts.
